# Ancestral social environments plus nonlinear benefits can explain cooperation in human societies

**DOI:** 10.1038/s41598-022-24590-y

**Published:** 2022-11-24

**Authors:** Nadiah P. Kristensen, Hisashi Ohtsuki, Ryan A. Chisholm

**Affiliations:** 1 Department of Biological Sciences, National University of Singapore, 16 Science Drive 4, Singapore, 117558 Singapore; 2 Department of Evolutionary Studies of Biosystems, School of Advanced Sciences, SOKENDAI, Shonan Village, Hayama, Kanagawa 240-0193 Japan; 3 Research Center for Integrative Evolutionary Science, School of Advanced Sciences, SOKENDAI, Shonan Village, Hayama, Kanagawa 240-0193 Japan

**Keywords:** Evolution, Evolutionary theory, Social evolution

## Abstract

Human cooperation (paying a cost to benefit others) is puzzling from a Darwinian perspective, particularly in groups with strangers who cannot repay nor are family members. The beneficial effects of cooperation typically increase nonlinearly with the number of cooperators, e.g., increasing returns when cooperation is low and diminishing returns when cooperation is high. Such nonlinearity can allow cooperation between strangers to persist evolutionarily if a large enough proportion of the population are already cooperators. However, if a lone cooperator faces a conflict between the group’s and its own interests (a social dilemma), that raises the question of how cooperation arose in the first place. We use a mathematically tractable evolutionary model to formalise a chronological narrative that has previously only been investigated verbally: given that ancient humans interacted mostly with family members (genetic homophily), cooperation evolved first by kin selection, and then persisted in situations with nonlinear benefits as homophily declined or even if interactions with strangers became the norm. The model also predicts the coexistence of cooperators and defectors observed in the human population (polymorphism), and may explain why cooperators in behavioural experiments prefer to condition their contribution on the contributions of others (conditional cooperation in public goods games).

## Introduction

How did humans evolve the ability to cooperate in groups with strangers? Social situations often involve a conflict between the group’s interests and an individual’s interests, i.e., they are social dilemmas^1^, and paying a cost to benefit others is puzzling from a ‘selfish’ Darwinian perspective. Nevertheless, humans not only regularly cooperate with strangers in daily life, they even cooperate in lab-based games that have been specifically designed to ensure the conflict^2^. In a lab-based linear public goods game (PGG), anonymous participants (i.e., mutual strangers) can contribute money to a common pool, which is then multiplied and split between them regardless of who contributed. The multiplier is chosen such that the group’s payoff is maximised when everyone contributes, but an individual’s payoff is maximised by not contributing; yet consistently, some participants will contribute^3,4^.

**Figure 1:**
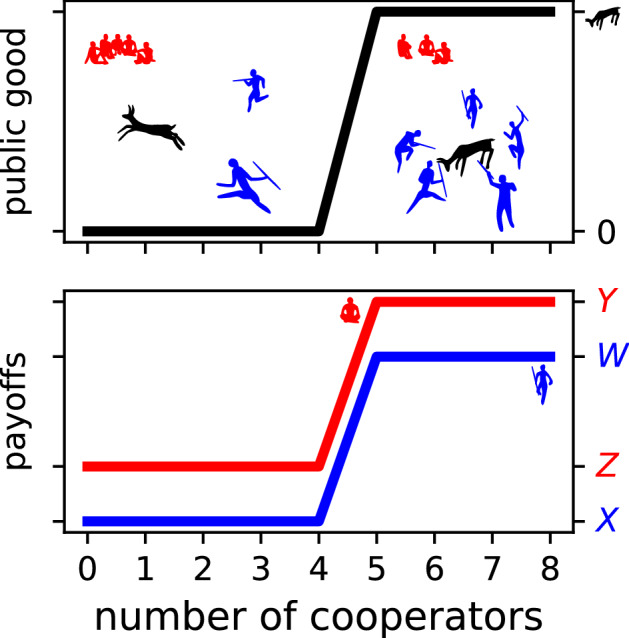
A hypothetical threshold public goods game involving a prehistoric hunt (for modern examples, see^21,22^). A minimum number of hunters ($\tau = 5$) must cooperate to successfully surround and kill an animal or their efforts are wasted. A cooperator’s payoff is *W* if the threshold is met and *X* if it is not (blue line). All members share the meat ($n=8$); therefore, the highest payoff goes to defectors (red line) regardless of whether the hunt is successful (payoff *Y*) or not (payoff *Z*). However, if an individual is likely to be the pivotal hunter, i.e., the hunter that brings the group above the threshold for a successful hunt, then it is incentivised to cooperate because the payoff to a defector when the threshold is not met is less than the payoff to a cooperator when the threshold is met ($Z < W$).

One possible resolution to this puzzle is that most real-life PGGs are in fact not linear^5–7^, and because cooperating with strangers in a nonlinear PGG can maximise individual payoff, individuals learn the social heuristic to cooperate and apply it to all seemingly similar PGGs^8–10^. The lab-based games above are ‘linear’ because there is a linear relationship between the level of contribution and the benefit from the public good (constant multiplier). In a linear PGG, when an individual switches from defect to cooperate, that individual’s payoff decreases by the same amount regardless of the number of cooperators in the game. Thus, the strength of the social dilemma is independent of cooperator frequency. However, if the benefit from the public good varies as a nonlinear function of the number of cooperators, then the switching gains may vary such that—at certain cooperator frequencies—the social dilemma is absent entirely. For example, in the threshold PGG (Fig. 1), a public good is provided when the number of cooperators in the group meets a minimum threshold^5,11,12^. Once the threshold is achieved, no defector has an incentive to cooperate, but nor will any cooperator switch to defect, because switching would cause the loss of the public good and thus decrease the former cooperator’s own payoff ($Z < W$ in Fig. 1)^13^. This game-theoretic analysis of the switching gains translates mathematically to the evolutionary dynamics^14^: in an infinitely large, well-mixed population, if the population already has a sufficiently large proportion of cooperators, then cooperation may persist. Thus, such nonlinear PGGs may be both a source of the social heuristics that misfire in lab-based linear PGGs^15^ and also explain how cooperation persists evolutionarily.

However, while nonlinearity may explain the evolutionary persistence of cooperation, cooperating is only adaptive if a large enough proportion of the population are already cooperators, which raises the question of how cooperation arose in the first place^6,16,17^. Rare cooperative ‘mutants’ can only invade a population of defectors if the payoff to a lone cooperator is higher than the payoff it would have received if it had defected instead^14^. In other words, cooperation can never evolve in a nonlinear PGG that presents a social dilemma to a lone cooperator (in contrast to, e.g., a by-product mutualism^18^).

One escape is if groups in the past tended to form between family members, i.e., genetic homophily, allowing cooperators to first invade by kin selection. Once cooperators built up sufficient numbers in the population, grouping with family was no longer necessary; cooperation can persist in certain nonlinear games due to their dynamical properties discussed above, even as the social environment shifts from interacting with kin to nonkin. The verbal argument that the invasion and persistence of cooperation in one-shot games can arise via a combination of kin selection and nonlinear benefits has been made before^16^, and some simulation results also support this intuition^6,17^, but a mathematically tractable model is needed to provide a rigorous basis for this narrative. One challenge is to account for genetic association between group members beyond pairs of individuals (Hamilton’s *r*), i.e., triplets, quadruplets, and so on (reviewed in^19^).

Here, we model how genetic homophily affects the evolutionary invasion and persistence of cooperation in such a nonlinear PGG. We treat the level of homophily as an extrinsic factor that reflects the prevailing social environment at a given point in time. To analyse the model, we use the higher-order genetic association approach of Ohtsuki^20^, which provides a systematic mathematical framework to account for genetic association between more than two group members. Our main technical innovation is to detail three different homophilic group-formation models, which allows us to obtain analytical results for the evolutionary dynamics. This facilitates easy identification of bifurcation points and regions of parameter space in which cooperation and defection are both evolutionarily stable, with the current state depending on the history of the system. We show that homophily can indeed facilitate the invasion of cooperation, and moreover, that cooperation can persist if homophily subsequently declines, in some cases even if homophily disappears altogether. Thus, we provide a rigorous explanation for how cooperation arose and persisted as humans expanded their social networks from kin to nonkin and strangers.

## Methods

We focused on the threshold PGG (e.g., Fig. 1), which is the nonlinear PGG that provides the clearest example of the evolutionary properties of interest (i.e., cooperators can persist but cannot invade). Previous authors show that the dynamical qualities of the threshold game are preserved in more general sigmoid games^14,16,17,23,24^, and we also obtain qualitatively similar results under homophily (see Supplementary).

In the threshold PGG, the public good is produced when the number of Cooperators in a group of size *n* meets or exceeds a threshold $\tau$, where $1< \tau < n$^5,11,12^. We defined the payoff structure with four parameters: *W* and *Y* are the payoffs to cooperators and defectors, respectively, if the threshold is met; and *X* and *Z* are the corresponding payoffs if the threshold is not met (Table 1). We imposed three constraints: (1) $\text {min}(W, X, Y, Z) = X$, which represents the risk of cooperation; (2) $Z<W$, which combined with the first constraint (in particular, $X < Z$) ensures that just meeting the threshold is Pareto optimal; and (3) $Z < Y$, which means Defectors benefit from the public good without contributing (free-riding). The constraints can be summarised as $X<Z, Z<W, Z<Y$. These constraints offered more flexibility than the one-parameter model common in the theoretical literature (e.g., $W = 1-c$, $X=-c$, $Y=1$, $Z=0$)^25,26^, reflecting the greater range explored in the experimental literature^27–29^.

**Table 1:** The payoffs that Cooperators and Defectors receive in the threshold PGG depending on whether or not the minimum threshold number of Cooperators ($\tau$) is met. Payoff parameters are subject to constraints: $X < Z$, $Z < W$, $Z < Y$.

	Threshold met	Threshold not met
Cooperator payoff	*W*	*X*
Defector payoff	*Y*	*Z*

To characterise the evolutionary dynamics, we used the higher-order genetic association approach of Ohtsuki^20^, which quantifies the change in the proportion of Cooperators in the population over time, $\Delta p$ (Supplementary S1). The model assumes an infinitely large population of haploid individuals, and we refer to subsets of individuals that are identical by descent as “families”. Each individual has a genetically determined pure Cooperate or Defect strategy, and its payoff from the threshold game determines the number of clonal offspring it contributes to the next generation. The model assumes that all offspring join a global pool, so competition in this model is global and there is no local competition^30^. Our approach is equivalent to the replicator-dynamics approach under random group formation, but it can also account for the genetic association between group members under homophilic group formation. Homophily is imposed as an extrinsic factor in our model that increases the probability that kin group together, and thus indirectly increases the probabilities that Cooperators group together and Defectors group together. This modifies the probability distribution of outcomes, and thus the expected payoff given a particular strategy, and therefore influences $\Delta p$.

**Figure 2:**
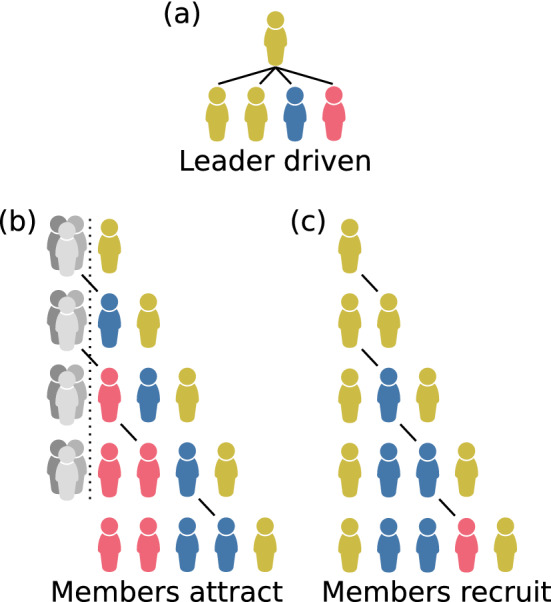
Examples of a group of five individuals forming according to the rules of the three homophilic group-formation models: (**a**) leader driven, (**b**) members attract, and (**c**) members recruit. Current members sequentially recruit or attract new members (lines) who tend to be kin (same colour). The grey group in (**b**) represents attraction of nonkin to the group itself.

Defining a homophilic group-formation process allows us to derive the probability distribution of different group compositions—i.e., how the group is divided into families that are identical by descent, and thus share the same strategy—and therefore we can obtain analytic expressions for $\Delta p$. We explored three different homophilic group-formation models (details in Supplementary S2) where groups are formed sequentially and current members tend to recruit or attract kin (Fig. 2). These models were chosen primarily as plausible simplifications of reality, but also selected because they are different enough to potentially have different qualitative effects on the model (Supplementary S8). We defined the homophily level $h = [0,1]$ as the expected proportion of new recruits that are kin of an existing member. We are interested in the effect of a changing tendency to group with kin over time, with this tendency being imposed extrinsically and attributable to a changing social organisation that we do not explicitly model. Therefore, we varied the homophily level by treating the parameters describing kin recruitment as control parameters. The three group-formation models are as follows:

1. *Leader driven*: The leader is chosen at random from the population. The leader recruits/attracts nonkin with probability $q \in [0,1]$ and kin with probability $1-q$, and therefore $h \equiv 1-q$. This model was inspired by foraging-band formation among Nambikwara people (Brazil), where the group leader provides the focal point around which the group forms^31^.
2. *Members attract*: The initial member is chosen at random. Current group members have equal weighting 1 of attracting a new member who is kin, but nonkin members are also attracted to the group itself with collective weighting $\alpha \in [0, \infty )$, and therefore $h \equiv \left( \sum _{i=1}^{n-1} i/(\alpha +i) \right) /\left( n-1 \right)$ represents the expected proportion of kin recruited during group formation. This model was chosen because it describes a plausible mechanism while also providing a relatively simple description of the family partition probabilities, i.e., Ewens’ sampling formula^32^.
3. *Members recruit*: The initial member is chosen at random. Current group members have an equal chance to recruit the next member. Members recruit nonkin with probability $q \in [0,1]$ and kin with probability $1-q$, and therefore $h \equiv 1-q$. This model was created as an alternative to the members-attract model and represents an opposite extreme of which member drives recruitment to the leader-driven model.

## Results

If groups are formed randomly, Cooperators can never invade a population of Defectors because the payoff to a lone Cooperator in a group of Defectors is lower than the payoff to Defectors (Supplementary S4; e.g., Fig. S1). However, if group formation is sufficiently homophilic, Cooperators can invade.

**Figure 3:**
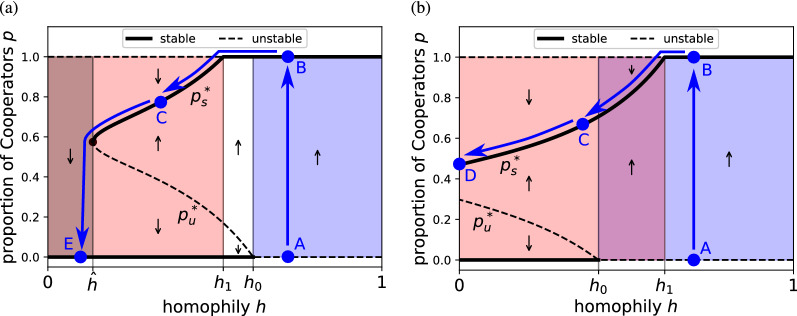
Two examples of how genetic homophily affects the evolutionary dynamics in our model, showing possible trajectories of cooperation as human homophily (tendency to interact with genetically related others) decreased over time due to changing social environments (blue lines). The evolutionary dynamics separates into qualitatively different regimes depending on the homophily level: $h < {\hat{h}}$, Cooperators cannot persist (dark shading); $h < h_1$, Defectors can both invade and persist (red shading); $h > h_0$, Cooperators can invade (blue shading). In the human ancestral past (e.g., $>1.6$ Ma^33^), homophily was high (point A), which under the model would have allowed cooperation to invade (B). As interactions with nonkin increased (*h* decreased), Cooperation persisted even into the region where it could not invade (C). Depending on the parameter values, Cooperation can either persist even if homophily disappears entirely (i.e., all interactions become nonkin; D) or be lost below a certain level of homophily (E). The example illustrated uses the members-recruit group-formation model ($h \equiv 1-q$) with parameter values $n=8$, $W=2$, $Y=3$, $Z=0$; (**a**) $\tau =5$, $X=-1$; (**b**) $\tau =4$, $X=-0.5$. See Supplementary S5 for qualitatively similar results from other group-formation models and for sigmoid PGGs.

Homophilic group-formation promotes cooperation by promoting both the invasion and persistence of Cooperators (Fig. 3 shows results for the members-recruit group-formation model; qualitatively similar results are obtained for other models, Supplementary S5). Mathematically, as homophily is increased from zero, three successive and biologically important bifurcations occur. First, when homophily is increased above some threshold ${\hat{h}}$ (Supplementary S6), the dynamics can transition from zero to two interior equilibria: an unstable polymorphism $p_u^*$ and a stable polymorphism $p_s^*$ (e.g., Fig. 3a). Therefore, Cooperators can coexist with Defectors under homophily where it was not possible under random group formation. Further increases in homophily move the unstable polymorphism towards the all-defect equilibrium ($p_u^* \downarrow$) and the stable polymorphism towards the all-cooperate equilibrium ($p_s^* \uparrow$). This both increases the proportion of Cooperators in the stable polymorphic population and lowers the initial proportion of Cooperators required to invade and persist. The second and third bifurcations occur when each of two interior equilibria collide with the corresponding trivial equilibria ($p_s^* \rightarrow p_1^*=1$ at $h=h_1$ and $p_u^* \rightarrow p_0^*=0$ at $h=h_0$, Supplementary S6), which causes the two trivial equilibria to flip between stable and unstable, which flips the invasibility of each strategy. For $h > h_1$, Defectors can no longer invade a population of Cooperators ($p_1^*$ stable), and for $h > h_0$, Cooperators can invade a population of Defectors ($p_0^*$ unstable).

Under random group formation, the stable coexistence of Cooperators and Defectors can be lost if the group size increases^26^ or the threshold $\tau$ is changed to an intermediate value^25^. Introducing homophilic group formation can reverse these effects and cause the stable polymorphism to reappear (Supplementary S7) in the same way as illustrated in Fig. 3a. The leader-driven group-formation model is the most conducive to cooperation, particularly at moderate group size (e.g., Supplementary Fig. S6): Defectors need lower homophily to invade and persist ($h_1$ is lower), Cooperators can invade at lower homophily ($h_0$ is lower), and Cooperators can persist at lower homophily (${\hat{h}}$ is lower). For the leader-driven and members-attract models, as the group size increases, the number of individuals in the group that are members of the largest family in the group asymptotes to a fixed proportion. In contrast, for the members-recruit model, the proportion asymptotes to 0, and therefore the members-recruit model is the least conducive to cooperation (Supplementary S8).

## Discussion

Over the course of human evolution, social interactions have expanded from kin to nonkin and strangers^34–38^, and that general trend continues today. For example, hominin material transport distances increased from $\sim$13 km before 1.6 Ma (comparable to chimpanzee home-range sizes) to hundreds of kilometres after 130 ka, indicating exchange networks^33^ and relationships beyond kin^39^. Today, hunter-gatherers maintain social networks of hundreds of unrelated individuals^40,41^ who congregate seasonally for communal hunting and social activities^22,42^, and in industralised societies, falling kin-network density is motivating new bases for social identity^43^.

When we imposed this pattern of declining genetic homophily on the results from our model, several chronological narratives emerged (blue arrows, Fig. 3). When homophily was high in the ancestral past, selection favoured cooperation, allowing the faculties that coordinate collective action to evolve (A $\rightarrow$ B, Fig. 3). The tendency to associate with kin declined over time, but cooperation was not necessarily lost. Cooperation persisted if a sufficiently high degree of homophily was retained (B $\rightarrow$ C), or if the game payoffs allowed cooperation to persist in the absence of homophily (C $\rightarrow$ D). In some cases in the model, the loss of homophily led to the loss of cooperation in that game (C $\rightarrow$ E), which cannot be recovered by simply reversing that incremental loss of homophily. Either homophily must be greatly increased to allow cooperation to reinvade (E $\rightarrow$ A $\rightarrow$ B), or a large proportion of Cooperators must invade the population together (above the separatrix, dashed line, Fig. 3).

Three main pieces of evidence suggest that our narrative is plausible. First, real-world benefits from hunting typically have sigmoid-like relationship with the number of hunters^17^, which qualitatively matches the shape of our model’s payoff functions. Collaborative foraging in general has been linked to the early stages of cooperation’s evolution^44^, including confrontational scavenging^45,46^, which also likely has a nonlinear benefit function^47^. Hunter-gatherers today often share meat among members of hunting groups formed on the basis of cultural rather than genetic kinship^21,42,48^, reflecting the later stages of our narrative. Second, if we are correct that cooperative social heuristics are derived from such real-life nonlinear PGGs, then participants in lab-based linear PGGs should behave as though they are playing nonlinear PGGs—and indeed they do. Cooperative participants prefer to condition their contributions on the contribution level of others^4,49,50^, which is only rational in nonlinear games. Conditional cooperation is also correlated with the belief that the payoff-maximising strategy depends on the contribution of others^51^, and chat logs from computer-networked games reveal a common misperception of linear PGGs as some type of coordination problem^52^. Third, the polymorphic coexistence of cooperators and defectors predicted by both our threshold game and qualitatively similar sigmoid games^14,16,17,23^ is consistent with the observation of polymorphism in human cooperative types^17^. Nonlinear games with a continuous strategy can also possess an interior equilibrium, i.e., a mixed strategy^53^, which is consistent with experimental participants’ tendency to avoid the extreme strategies of 100% defection or cooperation^15,54^.

Our model’s key contribution is to demonstrate the continuity of the evolutionary persistence of cooperation as interactions shifted from kin to strangers. The idea that cooperation first arose through kin selection is not new^16,55–57^, and some authors have emphasised that nonlinearity can permit cooperation to persist in one-shot PGGs with strangers without enforcement nor assortment^23^, but the connections between the two are typically explained verbally. A complete explanation for cooperation in social dilemmas must address both invasion and persistence of cooperative types^6^, and both past homophily and nonlinearity in our model are necessary: a linear game with past homophily may allow invasion but not persistence; and a nonlinear game without homophily may allow persistence but not invasion. We were able to obtain the necessary analytic expressions for the evolutionary dynamics (that could then be solved numerically) because we used the higher-order genetic association approach^20^. One barrier until now has been calculating family partition probabilities ($F_{n \rightarrow {\textbf{n}}}$), and our group-formation models may be of use to future workers (code and tutorials in Supplementary S9), including those who wish to build more realism into the models (e.g., Nambikwara leaders are not chosen at random but by reputation^31^).

We modelled homophilic group formation through direct recognition of family members, which in early hominins likely operated through co-residence duration^58^ and physical phenotype matching^59,60^, and later also through (culturally defined) kinship frameworks^61^; however, limited dispersal can also lead to homophilic group formation without kin recognition^62^. For example, Lehmann et al.^63^ explored the invasion and persistence of punishment using Wright’s infinite islands approach^64^, where the population is split into discrete patches with occassional dispersal of individuals between them. Although limited dispersal promotes homophily, it also promotes competition between kin, which can cancel out homophily’s positive effects on cooperation^65^. Therefore, the evolution of cooperation by limited dispersal typically requires some additional mechanism to export competition (reviewed in^66^), whereas offspring in our model always compete globally with offspring from other groups^30^. Several authors have shown that spatial clustering allows cooperation to invade threshold PGGs specifically^6,17^, and such spatially explicit lattice models can also reveal interesting, complex behaviour^67^; however, an analytical treatment of lattice models is difficult.

We wish to highlight some previous theoretical works with similar themes to our own. First, two conceptually similar papers modelled repeated interactions (in contrast to our one-shot game) with contingent strategies: threshold-triggered defection^57^ and also punishment^56^. They too found that high relatedness can facilitate the invasion of cooperative strategies that could otherwise only persist but not invade. In contrast to our model, they used the infinite-islands approach discussed above, and the cancellation effect of kin competition was averted by the additional assumption that population size on each ‘island’ was elastic (budding was also discussed). Thus, genetic assortment is present in both their and our approaches, and both export competition, but the scenario considered and assumptions needed differ. Second, Peña et al.^68^ studied the convergence stability of mixed strategies of discrete actions in nonlinear games. They assumed weak selection and that mutations caused only a small deviation in strategy, which allowed them to omit higher-order genetic associations (beyond dyadic relatedness). They found that a PGG with relatedness was equivalent to a game between randomly drawn players with rescaled payoffs (their Eq. 15). Consequently, the effects of relatedness in their model differ from those in our model, where our mutant invader plays a new discrete strategy (e.g., a Cooperator invading a population of Defectors) and the higher-order genetic associations must be accounted for. Finally, for a study of relatedness in nonlinear PGGs with continuous levels of investment (in contrast to our discrete strategies), we refer readers to Coder Gylling and Brännström^69^.

The two mechanisms that can promote cooperation between nonkin that have received the most attention in the literature—repeated interactions and reputation effects—involve violations of typical lab-based PGG assumptions (i.e., one-shot, anonymous interactions), and our model suggests the assumption of linearity also deserves scrutiny. Repeated interactions have a strong effect on cooperative behaviour in group games^70^, and may partly explain conditional cooperation in a 2-player games^71,72^; however, tit-for-tat like cooperative strategies can only persist in many-player linear PGGs under restrictive conditions^73^ (but see^74^). There is good evidence that reputational mechanisms influence cooperation within groups^70,75–78^; however, for reputation to serve as a complete explanation for cooperation in anonymous lab-based games, one must assume that the cue responses governing cooperation cannot easily distinguish between anonymous and reputation-relevant interactions, which is controversial^15,40,79^. In contrast, it seems plausible that humans have not evolved proximate mechanisms that can properly interpret payoff functions. Even in the conceptually simple threshold game, the calculation required to determine whether a cooperative symmetric Nash equilibrium exists is difficult^26,80^ (Supplementary S4.2.4), and experimental participants instead use a process of trial-and-error to converge on the equilibrum^12,28,81–83^. If most real-life PGGs are nonlinear, then relying on social heuristics is likely the only practical option available. Therefore, we agree with Raihani and Bshary^15^ that real-life nonlinear PGGs may be a significant source of social heuristics, and with Archetti et al. ^7^ that nonlinearity may be an important part of the explanation for cooperation between strangers.

Social heuristics provide a proximate explanation, and evolution an ultimate explanation, for why humans cooperate, and our model closes this gap for social dilemmas analogous to nonlinear PGGs. The gap remains open for other mechanisms of cooperation persistence; for example, Orma people have relatively high PGG contributions^3^ likely because their real-life public-goods practice includes punishment^84^, but how punishment first arose remains an open question^63,85–87^. In cultures with real-life monetary nonlinear PGGs, we may expect experimental participants to start with a modest expectation that cooperating will be payoff maximising and learn not to cooperate instead^88–90^. Combining social heuristics with an evolutionary approach may provide additional insights. For example, the positive assortment of cooperative Hadza campmates^91^ is probably due to social-learning dynamics rather than assortative preferences^92–94^. If each dry-season camp is roughly analogous to a single group-game in our model, then some years the camp will successfully converge on cooperation and some years it will not, which determines the ‘mood’ of camp members when they participate in the experiment (cf.,^95^), but cooperation persists in the population because it is not selected against in the long term.

Our model abstracts and simplifies the narrative, and we wish to highlight some key points where greater realism might be added in the future. Cooperative activities like hunting likely involved nontrivial cognitive faculties (e.g., joint intentionality^44^, communication abilities^33,96^, and understanding roles and expectations^97^) that provided a substrate for cooperation with nonkin—our model ignores cognitive evolution, but including it could enhance the persistence of cooperation. We also treated homophily level as an extrinsic parameter; however, it would be interesting to study a model where homophily is an intrinsic trait^98^. We speculate that the opportunity to cooperate with nonkin may have entered a positive feedback with declining homophily, increasing the number of potential cooperative partners and therefore the number of games played and benefits produced. We can contrast this with the evolutionary maladaption hypothesis^99–101^, where ancestral cooperation was indiscriminate but—due to limited dispersal—typically directed at kin, and was only extended to nonkin later and maladaptively due to e.g., rapidly increasing population size. In our model, individuals who cooperate with nonkin are not necessarily maladapted; therefore, their behaviour was not selected against. The key challenge would have been to overcome kin bias, potentially by developing new cues for cooperation (e.g., sharing a common fate^102,103^). The exact mechanisms (including cultural^104^) are outside the scope of our model, but we note that contemporary humans often cooperate with nonkin using cooptations of kin psychology^105–107^. For example, socially constructed notions of kinship^61,108^ coopt both the kinship framework that coordinates cooperation^109,110^ and the kin-psychological impulse to do so^111^. Interestingly, homophily in the broader sense of generalised attraction to similar others^112,113^ likely also has roots in kin psychology^105,114^, and the human tendency to empathise^115^ and cooperate with similar others even based on meaningless traits^116,117^ may have provided a starting point for nongenetic bases of group identity^118^, which functions by triggering the expectation that others will coordinate their cooperation with one’s own (cf.,^21,75^).

In conclusion, we propose that cooperation in human societies first arose due to kin selection, but as humans expanded their interactions to nonkin, it persisted due to the dynamical properties of certain nonlinear PGGs. Cooperative one-shot encounters with strangers occur frequently in modern society. We hope that as our understanding of what promotes or impedes such cooperation grows, we will become better equipped to tackle modern social and environmental dilemmas^119^.

## Supplementary Information

Supplementary Information.

## Data Availability

Functions and scripts used to generate the results, along with some tutorials, are archived in the Github repository: https://github.com/nadiahpk/homophilic-threshold-PGG, DOI: 10.5281/zenodo.7344398.
