# Supplementary material for: Ancestral social environments plus nonlinear benefits can explain cooperation in human societies
Source: Sci Rep. 2022 Nov 24;12:20252. doi: 10.1038/s41598-022-24590-y (PMC9691629; doi:10.1038/s41598-022-24590-y)
Supplement: Supplementary file 1 — Supplementary Information. [file 41598_2022_24590_MOESM1_ESM.pdf]

Ancestral social environments plus nonlinear benefits can explain cooperation in human societies

Supplementary Information

Nadiah P. KRISTENSEN, Hisashi OHTSUKI, Ryan A. CHISHOLM

S1 The higher-order genetic association approach

One technical challenge to modelling kin selection in nonlinear group games is that we must not only account for relatedness between pairs of individuals (Hamilton's r), but also between triplets, quadruplets, etc. [120–128] (reviewed in [19]). To achieve this, Ohtsuki [20] developed a mathematical framework. The following results in this section are from Ohtsuki [20].

We consider an infinitely large clonally reproducing population of haploid individuals, each with a genetically determined pure Cooperate or Defect strategy. Individuals form genetically homophilic groups to play an n-player game. Let a_k and b_k be the payoff functions for Cooperators and Defectors, respectively, when k of the other $n-1$ group members are Cooperators. In our threshold game, payoffs to Cooperators are and Defectors are

$$a_k = \begin{cases} W & \text{if } k \geq \tau - 1, \\ X & \text{otherwise,} \end{cases} \tag{S1a}$$

$$b_k = \begin{cases} Y & \text{if } k \geq \tau, \\ Z & \text{otherwise.} \end{cases} \tag{S1b}$$

Payoffs determine the number of offspring contributed to the next generation. Let ρ_l be the probability that all the l players randomly sampled from the group without replacement are Cooperators and set $\rho_0 = 1$. Then, the change in the proportion of Cooperators p in the population over one generation is [20]

$$\Delta p \propto \sum_{k=0}^{n-1} \sum_{l=k}^{n-1} (-1)^{l-k} \binom{l}{k} \binom{n-1}{l} \left[(1-\rho_1)\rho_{l+1} a_k - \rho_1(\rho_l - \rho_{l+1}) b_k \right]. \tag{S2}$$

Probabilities ρ_l depend on p and on the proportion of group members whose strategies are identical by descent (IBD). We define a "family" to be a group of individuals that are IBD. Let $\theta_{l\to m}$ be the probability that, if we draw l individuals without replacement from the group, they share exactly m common ancestors. Then [20]

$$\rho_l = \sum_{m=1}^{l} \theta_{l\to m} p^m. \tag{S3}$$

The higher-order genetic association, $\theta_{l\to m}$, depends on $F_{n\to\mathbf{n}}$, which are the probabilities that a group of size n has family partition structure $\mathbf{n}$, where $\mathbf{n}$ is a multiset of positive integers that sum to n. $F_{n\to\mathbf{n}}$ depends on the group-formation process.

In summary, to describe the evolutionary dynamics, we create a homophilic group-formation model (S2) to calculate all $F_{n\to\mathbf{n}}$, which we use to calculate $\theta_{l\to m}$ (S3), and ρ_l (Eq. S3), and combine with the threshold game payoff functions a_k and b_k (Eqs. S1) to describe the evolutionary dynamics (Eq. S2).

S2 Three homophilic group-formation models to calculate the probabilities of family partition structures, $F_{n\to\mathbf{n}}$

To calculate the probabilities of different family partition structures, $F_{n\to\mathbf{n}}$, we created three homophilic group-formation models: leader driven, members recruit, and members attract. Conceptually, we can imagine that leaders and members prefer to recruit or attract their own family members, and the homophily level determines the probability with which a 'mistake' is made and a nonkin member is recruited or attracted instead.

S2.1 Leader driven

Group formation is driven by the leader, who attracts or recruits kin with probability $1-q$ and nonkin with probability q (e.g., foraging-band formation, Nambikwara people, Brazil [31]).

Let $s = 0, \dots, n-1$ be the number of nonkin recruited. Because the population is infinite, every nonkin individual recruited will be from a new family to the group. The only possible family partition structures $\mathbf{n}_s$ have one family containing $n-s$ individuals and s families containing a solitary individual, i.e., $\mathbf{n}_0 = \{n\}$, $\mathbf{n}_1 = \{n-1, 1\}$, $\mathbf{n}_2 = \{n-2, 1, 1\}$, ..., $\mathbf{n}_{n-1} = \{1, 1, \dots, 1\}$. Therefore, the nonzero family partition probabilities are

$$F_{n\to\mathbf{n}_s} = \binom{n-1}{s} q^s (1-q)^{n-1-s}. \tag{S4}$$

For example, in Fig. 2a in the main text, $s = 2$ and $\mathbf{n}_s = \{3, 1, 1\}$.

S2.2 Members attract

At each step in the group-formation process, each current member attracts its own kin to join the group with weighting 1, and strangers are attracted to the group itself with weighting α. Because the population is infinite, the stranger will be from a new family to the group.

The jth recruit (or $(j+1)$-th group member) will be from a new family with probability

$$P(j \text{ from a new family}) = \frac{\alpha}{\alpha + j}, \tag{S5a}$$

or from a family a that has already been recruited to the group with probability

$$P(j \text{ from family } a) = \frac{n_a}{\alpha + j}, \tag{S5b}$$

where n_a is the number of current members from family a.

This model is equivalent to the sequential sampling scheme used in genetic coalescence models [129], and the partition probabilities are described by Ewens' formula [32]

$$F_{n\to\mathbf{n}} = \frac{n! \, \alpha^{\Phi}}{\prod_{j=1}^{n} j^{\phi_j} \phi_j! (\alpha + j - 1)}, \tag{S6}$$

where ϕ_j is the number of families with j members. For example, in Fig. 2c, $\mathbf{n} = \{1, 2, 2\}$ and $\phi = (1, 2)$.

In the main text, we have interpreted the α parameter in the members-attract group-formation model as attraction to the group itself. The connection to the neutral genetic coalescence model [129] is suggestive of another interpretation: the same distribution of family partition structures would also result from random sampling from a finite population with mutation rate α or subpopulation with immigration rate α under weak selection. However, this is not the interpretation we have in mind.

S2.3 Members recruit

S2.3.1 Overview

At each step in the group-formation process, a current member is chosen at random to be the recruitor. The recruitor recruits its own family member to the group with probability $1-q$, or recruits a stranger with probability q. Because the population is infinite, the stranger will be from a new family to the group.

The jth recruit (or $(j+1)$-th group member) will be from a new family with probability

$$P(j \text{ from a new family}) = q, \tag{S7a}$$

or from a family a that has already been recruited to the group with probability

$$P(j \text{ from family } a) = \frac{n_a(1-q)}{j}, \tag{S7b}$$

where n_a is the number of current members from family a.

Consider a particular arrival sequence of individuals $\mathbf{s}$ (e.g., Fig. 2c: $\mathbf{s} = (\text{yellow}, \text{yellow}, \text{blue}, \text{blue}, \text{pink})$). The arrival sequence implies a partition structure $\mathbf{n}$ (e.g., Fig. 2c: $\mathbf{n} = \{1, 2, 2\}$). If there are Φ families, then there were $\Phi - 1$ nonkin recruitments. Let $\mathbf{m} = (m_1, \ldots, m_{\Phi-1})$ be the ordinals of each nonkin recruitment (e.g., Fig. 2c: $\mathbf{m} = (2, 4)$). Then the probability of $\mathbf{s}$ is (see example in S2.3.2)

$$P(\mathbf{s}) = \left(\frac{\prod_{i=1}^{\Phi}(n_i - 1)!}{(n-1)!} q^{\Phi-1}(1-q)^{n-\Phi} \right) \prod_{k=1}^{\Phi-1} m_k. \tag{S8}$$

The left-hand bracketed factor is the same for any sequence that has partition structure $\mathbf{n}$. Therefore, to get the total probability $P(\mathbf{n}) = F_{n\rightarrow\mathbf{n}}$ that is our goal, we need to identify every $\mathbf{m}$ consistent with $\mathbf{n}$, and count the number of $\mathbf{s}$ consistent with $\mathbf{m}$. The mistake ordinals $\mathbf{m}$, however, depend on the family arrival order $\vec{\mathbf{n}}$, so the sum is easier to obtain in the two steps described below.

Define $\vec{\mathbf{n}}$ as a multiset permutation of $\mathbf{n}$ corresponding to family arrival orders (e.g., Fig. 2c: $\vec{\mathbf{n}} = (2, 2, 1)$). Define $\mathcal{N}$ as the set of all $\vec{\mathbf{n}}$ consistent with $\mathbf{n}$. For example, if $\mathbf{n} = \{1, 2, 2\}$, then $\mathcal{N} = \{(1, 2, 2), (2, 1, 2), (2, 2, 1)\}$. Define $\mathcal{M}$ as the set of all $\mathbf{m}$ consistent with $\vec{\mathbf{n}}$, which is the set of all $\mathbf{m}$ satsifying the constraints $m_{i-1} + 1 \leq m_i \leq \hat{m}_i$ where $m_0 = 0$ and $\hat{m}_i = \sum_{k=1}^{i} \vec{n}_k$. For example, if $\vec{\mathbf{n}} = (1, 2, 2)$, then $\mathcal{M} = \{(1, 2), (1, 3)\}$ (another, larger, example is provided in S2.3.3). Call the count of possible individual arrival sequences $\mathbf{s}$ consistent with $\vec{\mathbf{n}}$ and $\mathbf{m}$ as $C(\vec{\mathbf{n}}, \mathbf{m})$. Then

$$F_{n\rightarrow\mathbf{n}} = P(\mathbf{n}) = \left(\frac{\prod_{i=1}^{\Phi}(n_i - 1)!}{(n-1)!} q^{\Phi-1}(1-q)^{n-\Phi} \right) \sum_{\vec{\mathbf{n}}\in\mathcal{N}} \sum_{\mathbf{m}\in\mathcal{M}} C(\mathbf{m}, \vec{\mathbf{n}}) \prod_{k=1}^{\Phi-1} m_k. \tag{S9}$$

To count $C(\vec{\mathbf{n}}, \mathbf{m})$, we fix the family identity of the first individual and every recruitment mistake, and work through the families in the reverse order of arrival, counting how many ways there are to arrange individuals from that family given the positions taken up by families that arrive later (see worked example in S2.3.4). Using this approach, we obtain

$$C(\mathbf{m}, \vec{\mathbf{n}}) = \prod_{j=2}^{\Phi} \binom{\hat{m}_j - m_{j-1} - 1}{\vec{n}_j - 1}. \tag{S10}$$

Substituting Eq. S10 into Eq. S9 gives the final expression

$$F_{n\rightarrow\mathbf{n}} = \left(\frac{\prod_{i=1}^{\Phi}(n_i - 1)!}{(n-1)!} q^{\Phi-1}(1-q)^{n-\Phi} \right) \sum_{\vec{\mathbf{n}}\in\mathcal{N}} \sum_{\mathbf{m}\in\mathcal{M}} \prod_{j=2}^{\Phi} \binom{\hat{m}_j - m_{j-1} - 1}{\vec{n}_j - 1} \prod_{k=1}^{\Phi-1} m_k. \tag{S11}$$

S2.3.2 Example: Find $P(\mathbf{s})$

What is the probability $P(\mathbf{s})$ of the sequence

$$\mathbf{s} = a\ a \mid b\ a \mid c\ b\ c\ c\ a\ b\ b \tag{S12}$$

where $a, b, c,$ identify the family memberships of individuals, the order of the letters signifies the order in which the individuals joined the group, and recruitment mistakes are highlighted with a '|'?

From the description of the group-formation model in Eq. S7

$$
\begin{aligned}
P(\mathbf{s}) &= (1-q)\left(\frac{2q}{2}\right)\left(\frac{2(1-q)}{3}\right)\left(\frac{4q}{4}\right)\left(\frac{1-q}{5}\right)\left(\frac{1-q}{6}\right)\left(\frac{2(1-q)}{7}\right)\left(\frac{3(1-q)}{8}\right) \\
&\qquad \left(\frac{2(1-q)}{9}\right)\left(\frac{3(1-q)}{10}\right) \\
&= \left(\frac{\overbrace{(3!)(1-q)^3}^{a}\ \overbrace{(3!)(1-q)^3}^{b}\ \overbrace{(2!)(1-q)^2}^{c}}{(n-1)!}\right)\underbrace{\left((2)(4)q^2\right)}_{\text{mistakes}} \\
&= \left(\frac{(3!)(3!)(2!)}{(n-1)!}\right)\left(q^2(1-q)^8\right)\left((2)(4)\right). \qquad \text{(S13)}
\end{aligned}
$$

Eq. S13 is written in a way to illustrate the connection to Eq. S8. The denominator in the first bracketed term corresponds the number of recruitments, which is the group size minus 1, i.e., $n-1$. The factorials in the numerator correspond to the family partition structure, i.e., $\mathbf{n} = \{4, 4, 3\}$. The second bracketed term depends the number of recruitments of nonkin and kin: there are $\Phi = 3$ families in the group, which implies 2 nonkin, which gives q^2; and $n-1-(\Phi-1) = 8$ kin gives $(1-q)^8$. The final bracketed term is the product of the mistake ordinals, $\mathbf{m} = (2, 4)$.

S2.3.3 Example: Find the constraints on $\mathbf{m}$ corresponding to $\vec{\mathbf{n}}$

If a group has family arrival order $\vec{\mathbf{n}} = (4, 5, 2, 2)$, what are the constraints on the values that the mistake ordinals can take, $\mathbf{m} = (m_1, m_2, m_3)$?

Denote the family memberships in $\vec{\mathbf{n}}$ by (a, b, c, d). The first individual is not recruited; therefore, the first recruitment mistake m_1 corresponds to the arrival of the first individual from the second family, family b.

The earliest that the first mistake can be is on the first recruitment, i.e., $1 \leq m_1$. Once the first mistake has occurred, the earliest that the second mistake can be is immediately after the first mistake, i.e., $m_1 + 1 \leq m_2$. Therefore, in general, $m_{i-1} + 1 \leq m_i$ where we define $m_0 = 0$.

The latest that each mistake can occur happens in the sequence where every family arrives as a group. For the example above, that is the sequence $\mathbf{s} = a\,a\,a\,a \mid b\,b\,b\,b\,b \mid c\,c \mid d\,d$. Therefore, in general, $m_i \leq \hat{m}_i$ where $\hat{m}_i = \sum_{k=1}^{i} \vec{n}_k$.

For the example above, the constraints are:

$$
\begin{aligned}
1 \leq m_1 &\leq 4 \\
m_1 + 1 \leq m_2 &\leq 9 \\
m_2 + 1 \leq m_3 &\leq 11
\end{aligned}
$$

S2.3.4 Expression for $C(\vec{\mathbf{n}}, \mathbf{m})$

How many individual arrival sequences are consistent with a family arrival order $\vec{\mathbf{n}} = (4, 5, 2, 2)$ and recruitment-mistake ordinals $\mathbf{m} = (1, 5, 8)$?

One possible arrival order of individuals that satisfies $\vec{\mathbf{n}}$ and $\mathbf{m}$ above is

$$a \mid b\,a\,b\,b \mid c\,a\,b \mid d\,a\,c\,b\,d \qquad \text{(S14)}$$

Arrival sequences consistent with $\vec{\mathbf{n}}$ and $\mathbf{m}$ rearrange the letters above with the constraints that: (1) the first a is fixed; (2) every letter after a '|' is fixed; and (3) letters can only be placed after their corresponding '| x'.

Start at the end with family d, the fourth ($j = 4$) family to join the group. There are four free positions into which one individual must be positioned:

$$a \mid b\,a\,b\,b \mid c\,a\,b \mid d\ \underbrace{a\,c\,b\,d}_{\circ\,\circ\,\circ\,d}. \qquad \text{(S15)}$$

The number of ways to make this choice is $\binom{4}{1} = 4$.

Consider family c, the third ($j = 3$) family to join. Two positions are already occupied by family d members, so there are five free positions into which one individual must be positioned:

$$a \mid b\,a\,b\,b \mid c\,\underbrace{a\,b \mid d\,a\,c\,b\,d}_{\circ\,\circ \mid \times\,\circ\,c\,\circ\,\times}. \tag{S16}$$

The number of ways to make this choice is $\binom{5}{1} = 5$.

Consider family b, the second ($j = 2$) family to join. Four positions are already occupied by family c and d members, so there are seven free positions into which four individuals must be positioned:

$$a \mid b\,\underbrace{a\,b\,b \mid c\,a\,b \mid d\,a\,c\,b\,d}_{\circ\,b\,b \mid \times\,\circ\,b \mid \times\,\circ\,\times\,b\,\times}. \tag{S17}$$

The number of ways to make this choice is $\binom{7}{4} = 35$.

Taking the product of the combinations, the total number of possible arrival sequences is

$$C(\mathbf{m}, \vec{\mathbf{n}}) = 4 \times 5 \times 35 = 700. \tag{S18}$$

The example above helps us generalise. In general, for each family j considered above, the number of free positions was

$$n - m_{j-1} - 1 - \sum_{k=j+1}^{\Phi} \vec{n}_k = \hat{m}_j - m_{j-1} - 1 \tag{S19}$$

and the number of individuals to be positioned was

$$\vec{n}_j - 1. \tag{S20}$$

Therefore, we can write the general equation

$$C(\mathbf{m}, \vec{\mathbf{n}}) = \prod_{j=2}^{\Phi} \binom{\hat{m}_j - m_{j-1} - 1}{\vec{n}_j - 1} \tag{S21}$$

S3 How to find the elements of matrix M, which is used to calculate higher-order genetic associations θ from family partition-structure probabilities $\mathbf{F}$

S3.1 Overview

Ohtsuki [20] (Appendix C) provided an example for group size $n = 4$ of how to calculate the higher-order genetic associations $\theta_{l\to m}$ from the probabilities of different family partition structures $F_{n\to\mathbf{n}}$. Here, we provide the general method.

Define column vectors $\boldsymbol{\theta} = [\theta_{1\to 1}, \theta_{2\to 1}, \theta_{2\to 2}, \ldots, \theta_{n\to n}]^T$ and $\mathbf{F} = [F_{n\to\{1,\ldots,1,1\}}, F_{n\to\{1,\ldots,1,2\}}, \ldots, F_{n\to\{n\}}]^T$. Then there is a matrix M such that

$$\boldsymbol{\theta} = M\mathbf{F}. \tag{S22}$$

Call $M(l, m, \mathbf{n})$ the entry of M in the row corresponding to $\theta_{l\to m}$ and the column corresponding to $F_{n\to\mathbf{n}}$, which is the probability that a random sample of l individuals from a group with the family partition structure $\mathbf{n}$ will contain m families. For a given $\mathbf{n}$, index the entries in an arbitrary order, $\vec{\mathbf{n}} = (n_1, n_2, \ldots, n_\Phi)$, where n_i is the number of individuals in the group who are IBD with common ancestor i, and Φ is the total number of ancestors. Let $\mathcal{I}$ be the set of all $\mathbf{i} = (i_1, \ldots, i_m)$, which are m-length combinations of integers $1, \ldots, \Phi$. For example, if $\Phi = 4$ and $m = 3$, then $\mathcal{I} = \{(1,2,3), (1,2,4), (1,3,4), (2,3,4)\}$. Let $\mathcal{Q}$ be the set of all $\mathbf{q} = (q_{i_1}, \ldots, q_{i_m})$, which are multiset permutations of m-length partitions of l. For example, if $l = 5$ and $m = 3$, then $\mathcal{Q} = \{(1,1,3), (1,3,1), (3,1,1), (1,2,2), (2,1,2), (2,2,1)\}$. Then, the entries of M are

$$M(l, m, \mathbf{n}) = \left(\sum_{\mathbf{i}\in\mathcal{I}} \sum_{\mathbf{q}\in\mathcal{Q}} \prod_{j=1}^{m} \binom{n_{i_j}}{q_{i_j}} \right) \Big/ \binom{n}{l} \tag{S23}$$

S3.2 Explanation

Consider the outcome of randomly sampling $l \leq n$ individuals from a group with structure $\mathbf{n}$. The distribution of outcomes follows the multivariate hypergeometric distribution

$$P(Q_1 = q_1, Q_2 = q_2, \ldots, Q_w = q_w \mid n_1, n_2, \ldots, n_\Phi) = \frac{\prod_{i=1}^{\Phi} \binom{n_i}{q_i}}{\binom{n}{l}}, \tag{S24}$$

where q_i is the number of individuals in the sample from family i, and $\sum_i q_i = l$.

If $q_i = 0$, then $\binom{n_i}{q_i} = 1$, so we can simplify Eq. S24 by considering only those families that contribute to the product. Define

$$\mathbf{q} = (q_{i_1}, q_{i_2}, \ldots, q_{i_m}), \tag{S25}$$

where $\mathbf{i} = (i_1, \ldots, i_m)$ are the indices of the families that were sampled. Then

$$P(\mathbf{Q} = \mathbf{q} \mid \mathbf{n}) = \frac{\prod_{j=1}^{m} \binom{n_{i_j}}{q_{i_j}}}{\binom{n}{l}}. \tag{S26}$$

Eq. S26 gives the probability of drawing just one set of families $\mathbf{i}$ with distribution $\mathbf{q}$. To find the probability $M(l, m, \mathbf{n})$, we must take the sum over all possible families $\mathbf{i}$ and distributions $\mathbf{q}$ that satisfy (l, m) given $\mathbf{n}$. That

sum is

$$M(l,m,\mathbf{n}) = \binom{n}{l}^{-1} \left(\sum_{i_1=1}^{\Phi-m+1} \sum_{i_2=i_1+1}^{\Phi-m+2} \cdots \sum_{i_g=i_{g-1}+1}^{\Phi-m+g} \cdots \sum_{i_m=i_m+1}^{m} \right.$$
$$\left. \sum_{q_{i_1}=1}^{l-(m-1)} \sum_{q_{i_2}=1}^{l-(m-2)-q_{i_1}} \cdots \sum_{q_{i_h}=1}^{l-(m-h)-\sum_{j=1}^{i_{h-1}} q_{i_j}} \cdots \sum_{q_{i_{m-1}}=1}^{l-1-\sum_{j=1}^{i_{m-2}} q_{i_j}} \prod_{j=1}^{m} \binom{n_{i_j}}{q_{i_j}} \right), \quad \text{(S27)}$$

where $q_{i_m} = l - \sum_{j=1}^{i_{m-1}} q_{i_j}$.

Eq. S27 can be written more compactly as Eq. S23 above by replacing the nested sums with counts over the sets of i_j and q_{i_j}.

The i_j indices in Eq. S23 are all m-length combinations of integers $1, \ldots, \Phi$. For example, if there are $\Phi = 4$ families in the group, and the sample has $m = 3$ families, then the indices $\mathbf{i}$ are:

i_1	i_2	i_3
1	2	3
1	2	4
1	3	4
2	3	4

This can be found quickly in Python with: `itertools.combinations(range(Phi), m)`

The q_{i_j} values in Eq. S23 are all multiset permutations of m-length partitions of l. For example, if we draw $l = 5$ individuals, and we are interested in possible numbers of individuals in $m = 3$ groups, then the m-length partitions of l are $(1,1,3)$ and $(1,2,2)$, and their multiset permutations $\mathbf{q}$ are:

p_1	p_2	p_3	q_{i_1}	q_{i_2}	q_{i_3}
1	1	3	1	1	3
			1	3	1
			3	1	1
1	2	2	1	2	2
			2	1	2
			2	2	1

The unique permutations of a partition can be found with:
`sympy.utilities.iterables.multiset_permutations(partition)`.

To save computational time in our code (see S9 for an overview of the code repository), the partitions of all integers $l = 1, \ldots, n-1$ were found at the beginning of the process [130] and subsetted to match length m as needed. Most of the computational time is spent iterating through the nested loops, so identifying cases that can be skipped saves time:

1. The first row of M corresponding to $\theta_{1\to 1}$ is all ones.
2. In the last n rows of M, corresponding to $\theta_{n\to m}$, all elements are zeros except when $m = \Phi$, which are ones.
3. Entries of M corresponding to $m > \Phi$ are zeros.
4. Sort the group's partition $\mathbf{n}$ and sample's partition $\mathbf{p} \vdash l$ in reverse order. If any $n_i < p_i$ (for $i = 1, \ldots, m$), then for all $\mathbf{q}$ that are permutations of $\mathbf{p}$, $P(\mathbf{Q} = \mathbf{q} \mid \mathbf{n}) = 0$.

Matrices for games with group size up to $n = 24$ have been calculated and saved in the online code repository (S9). Please find a quickstart tutorial in `/tutorials/matrix_M.pdf` showing how to access and use these stored matrices.

S4 Dynamics under random group-formation model

S4.1 Evolutionary dynamics

Under random group formation (i.e., in the absence of homophily), Eq. S3 simply becomes $\rho_l = p^l$, and putting this into Eq. S2 yields the following replicator dynamics [131]

$$\frac{dp}{dt} = p(1-p)g(p) \tag{S28}$$

where $g(p)$ is known as the *gain function* [16]

$$g(p) = \sum_{k=0}^{n-1} \binom{n-1}{k} p^k (1-p)^{n-1-k} d_k \tag{S29}$$

where k is the number of the $n-1$ other members of the group that are Cooperators, and $d_k = a_k - b_k$ is the payoff gain if an individual switches from the Defector to Cooperator strategy. We define the *gain sequence* $\mathbf{d} = (d_0, d_1, \ldots, d_{n-1})$ for later use.

S4.2 Analysis of the dynamics

An inspection of Eq. S28 immediately reveals that there are two trivial equilibria, $p_0^* = 0$, a population of all Defectors; and $p_1^* = 1$, a population of all Cooperators. In our case, the Defector equilibrium $p_0^* = 0$ is always stable because $g(0) = d_0 = X - Z < 0$. On the other hand, $g(1) = d_{n-1} = W - Y$ suggests that the Cooperator equilibrium is stable if $W > Y$ and unstable if $W < Y$.

As for the internal equilibria of Eq. S28, the number of roots (counted with multiplicity) and sign changes in gain function, $g(p)$, is either equal to the number of sign changes in gain sequence, $\mathbf{d}$, or less by an even amount [Property 2 in 14]. This allows us to infer information about the existence of any interior equilibria, as detailed below.

Table S1: The payoff gain if an individual switches strategy from Defector to Cooperator, $d_k = a_k - b_k$, for the general case $1 < \tau < n$ assumed in the main text.

k	a_k	b_k	d_k	sgn(d_k)
0	X	Z	$X - Z$	$-$
$\vdots$	$\vdots$	$\vdots$	$\vdots$	$\vdots$
$\tau - 2$	X	Z	$X - Z$	$-$
$\tau - 1$	W	Z	$W - Z$	$+$
τ	W	Y	$W - Y$	$+$ or $-$
$\vdots$	$\vdots$	$\vdots$	$\vdots$	$\vdots$
$n - 1$	W	Y	$W - Y$	$+$ or $-$

S4.2.1 The case of $W > Y$

If $W > Y$, the sign pattern of the gain sequence is $\mathbf{d} = (-, \ldots, -, +, \ldots, +)$, which includes a single sign change, which in turn suggests a single internal equilibrium. By Result 3.2.a in [14], this interior equilibrium $0 < p_u^* < 1$ is unstable. Therefore, bistability occurs; the proportion of Cooperators must be above $p > p_u^*$ to establish and for the population to evolve to all-Cooperators; otherwise, it evolves to all-Defectors.

S4.2.2 The case of $W < Y$

If $W < Y$, the sign pattern of the gain sequence is $\mathbf{d} = (-, \ldots, -, +, -, \ldots, -)$, which includes two sign changes, which in turn suggests that $g(p)$ has zero or two roots (counted with multiplicity). In fact, Result 4.1 in [14] says that there may be zero, one, or two interior equilibria, depending on the maximum value of the gain function $g(\hat{p})$, as follows:

- If $g(\hat{p}) < 0$, there are zero interior equilibria. Therefore, the population always evolves to all-Defectors.
- If $g(\hat{p}) = 0$, there is one unstable interior equilibrium. This is a degenerate case.

- If $g(\hat{p}) > 0$, there are two interior equilibria $0 < p_u^* < p_s^* < 1$, where p_u^* is unstable and p_s^* is stable. Therefore, the proportion of Cooperators must be above $p > p_u^*$ to establish and for the population to evolve to a coexistence of Cooperators and Defectors (p_s^*); otherwise, it evolves to all-Defectors.

S4.2.3 Numerical examples

Fig. S1 illustrates the different regimes of the evolutionary dynamics in the baseline model with random group formation (no homophily). The key take-away is that the all-Defector $p^* = 0$ is always stable, and therefore Cooperators cannot invade a population of all Defectors.

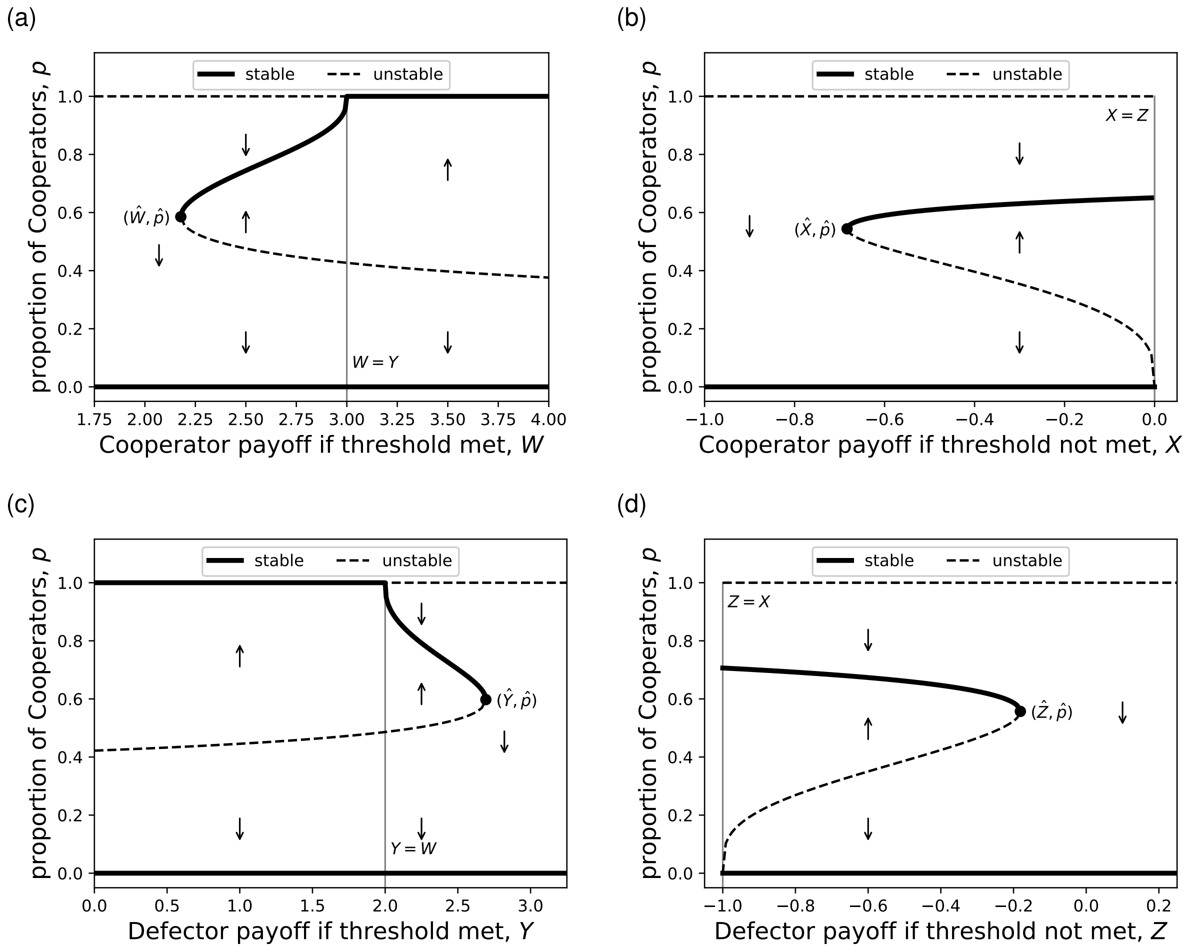

Figure S1: An example of the evolutionary dynamics of the baseline model, where groups are formed without homophily. Direction of selection is indicated by arrows; stable and unstable equilibria by thick lines and dashed lines, respectively; and the filled circle marks the point of transition from zero to two interior equilibria. Dynamics vary with game payoff parameters: (a) Cooperator payoff if threshold met W, (b) Cooperator payoff if threshold not met X, (c) Defector payoff if threshold met Y, (d) Defector payoff if threshold not met Z. The critical values that mark the transition from zero to two interior equilibria ($\hat{p}$, $\hat{W}$, $\hat{X}$, $\hat{Y}$, $\hat{Z}$) must be found numerically. Default parameter values: $n = 8$, $\tau = 5$, $W = 2$, $X = -1$, $Y = 3$, $Z = 0$.

S4.2.4 Critical point of transition from zero to two interior equilibria

The critical value that marks the transition from zero to two interior equilibria (filled circles in Fig. S1) occurs when the peak of the $g(p)$ function is touching 0 at a single point. Therefore, $\hat{p}$ simultaneously solves $g(\hat{p}) = 0$ and $g'(\hat{p}) = 0$. The derivative condition can be simplified to

$$\hat{p} = \frac{(\tau - 1)(\hat{W} - \hat{X})}{(\tau - 1)(\hat{W} - \hat{X}) + (\hat{Y} - \hat{Z})(n - \tau)}. \tag{S30}$$

In the typical one-parameter threshold game models analysed in the literature, which assume $W - X = Y - Z$, Eq. S30 reduces to $\hat{p} = (\tau - 1)/(n - 1)$ [25, 26]. The critical point is solved numerically (see `/scripts/random_grp/find_crit.py` for script).

S5 Similar results obtained from different models

The purpose of this section is to show that qualitatively similar evolutionary dynamics to those presented in the main text (Fig. 3) can also be obtained under different group-formation models and when the assumption of a binary threshold is relaxed to a sigmoid payoff function.

S5.1 Other group-formation models

Qualitatively similar dynamics are obtained under the leader-driven and members-attract group-formation models. Fig. S2 shows the results for the same group-size, threshold, and payoff parameter values as used in Fig. 3. The key result holds: increasing homophily promotes the invasion and persistence of Cooperators. We find again that a transition from zero to two interior equilibria occurs at $h > \hat{h}$ (Fig. S2a,c), that $p_s^* \uparrow$ and $p_u^* \downarrow$ as homophily increases, and that Cooperators can invade when $h > h_0$.

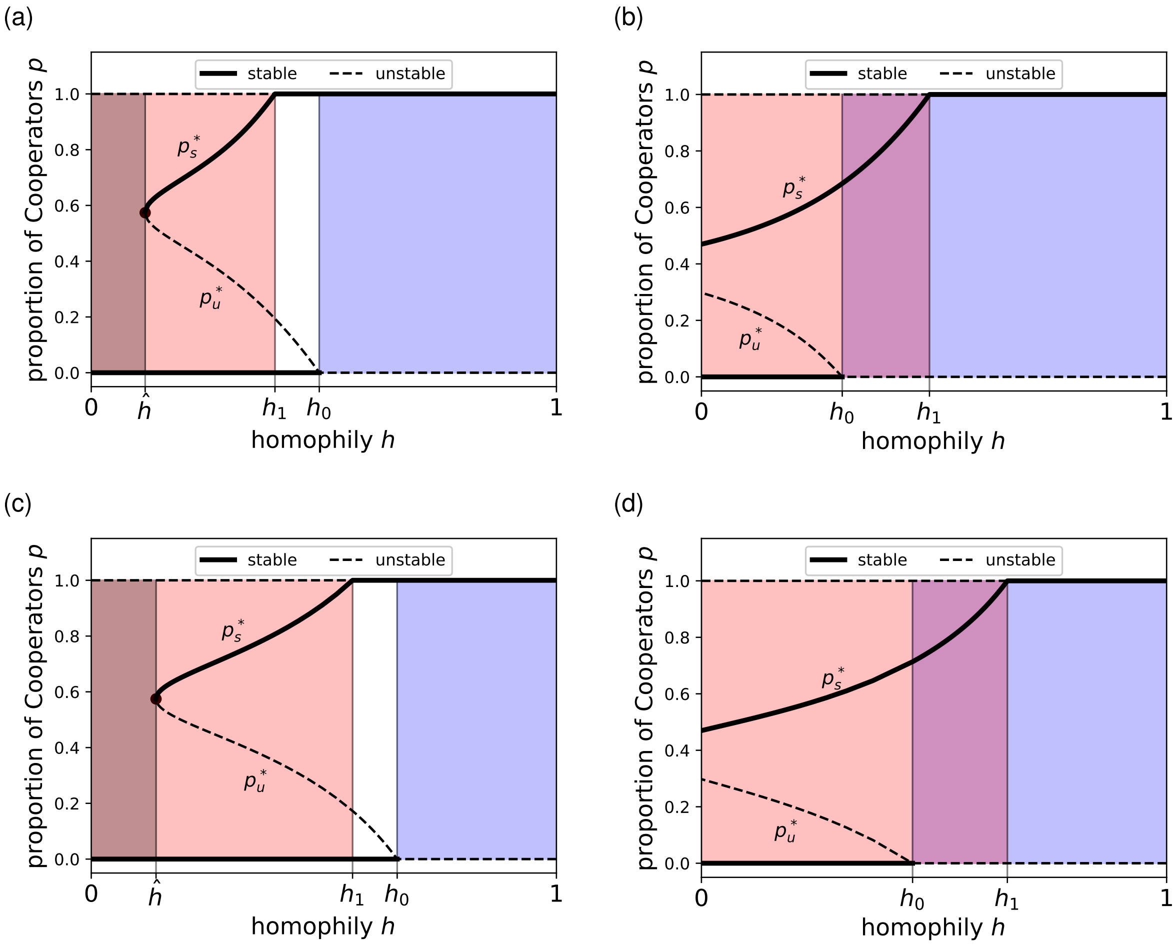

Figure S2: Examples of how homophily affects the evolutionary dynamics under the (a,b) leader-driven and (c,d) members-attract group-formation models. Parameter values are the same as in the members-recruit example in the main text (Fig. 3): $n = 8$, $W = 2$, $Y = 3$, $Z = 0$; (a,c) $\tau = 5$, $X = -1$; (b,d) $\tau = 4$, $X = -0.5$. Coloured regions separate regions of qualitatively different evolutionary dynamics: $h < \hat{h}$, Cooperators cannot persist (dark shading); $h < h_1$, Defectors can both invade and persist (red shading); $h > h_0$, Cooperators can invade (blue shading).

S5.2 Sigmoid payoffs

Previous authors have noted that the dynamical qualities of the threshold game are preserved under a wide range of games with sigmoid payoff functions [14, 16, 17, 23, 24], and we similarly found that the qualitative effects of homophily are preserved in sigmoid games.

Taking inspiration from Archetti [24], we replaced the payoff matrix (Table 1) with a more general nonlinear payoff function. Let k be the number of the other $n-1$ group members who are Cooperators. We define the payoff to Cooperators

$$a_k = X + (W - X)\frac{l_a(k) - l_a(0)}{l_a(n-1) - l_a(0)}, \quad \text{where} \quad l_a(k) = \frac{1}{1 + \exp\left(\frac{s(\tau - 1.5 - k)}{n-1}\right)}, \tag{S31}$$

and Defectors

$$b_k = Z + (Y - Z)\frac{l_b(k) - l_b(0)}{l_b(n-1) - l_b(0)} \quad \text{where} \quad l_b(k) = \frac{1}{1 + \exp\left(\frac{s(\tau - 0.5 - k)}{n-1}\right)} \tag{S32}$$

where s is a new parameter controlling the steepness of the sigmoid function. Varying s allows us to explore a spectrum of scenarios from the threshold game ($s \to \infty$) to the linear PGG ($s = 0$).

Fig. S3a-b show examples of the payoff functions when $s = 25$. Using the same parameter values as Fig. 3, the gain functions (S4 for details) indicate that the number of interior equilibria is preserved under random group formation (Fig. S3c-d). Increasing homophily also has the same effects as described in the main text, promoting the evolution of cooperation by promoting the invasion and persistence of Cooperators (Fig. S3e-f).

Decreasing the steepness parameter s moves the scenario closer to a linear PGG, where cooperation cannot persist. As expected, below a certain s, the gain sequence precludes the existence of interior equilibria, and Cooperators can no longer coexist with Defectors under random group formation (e.g., $s = 5$, Fig. S4). Nevertheless, the finding that homophily promotes the evolution of cooperation still holds: the dynamics transition from zero to two interior equilibria when $h > \hat{h}$, both interior equilibria move towards their respective axes as h increases, and Cooperators can invade a population of Defectors when $h > h_0$.

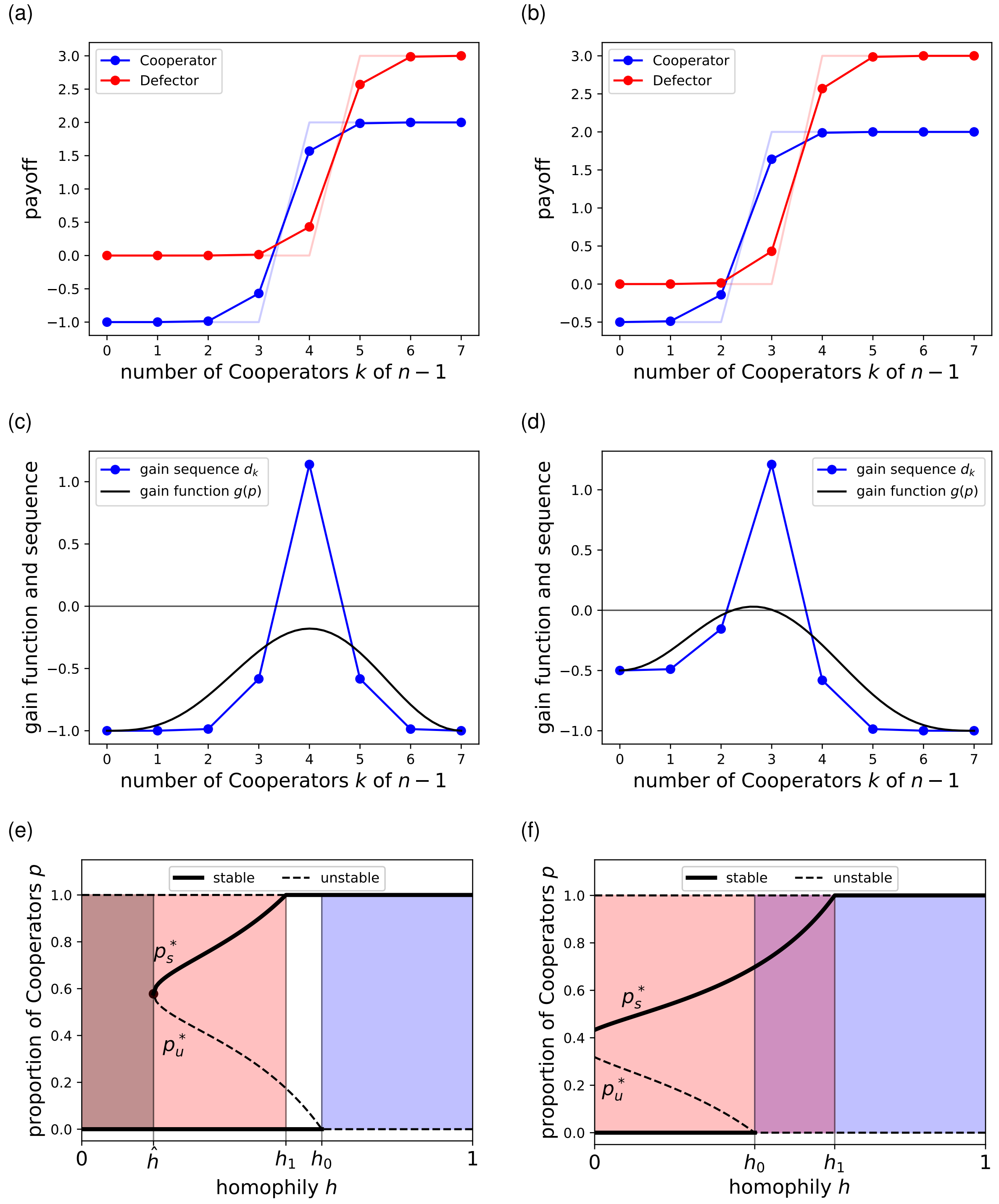

Figure S3: For comparison with Fig. 3 in the main text, two examples of how homophily affects the evolutionary dynamics in the sigmoid game with $s = 25$. (a-b) Cooperator and Defector payoff functions, with the threshold-game functions for comparison with opaque colours; (c-d) Gain sequence and gain function; and (e-f) summary of evolutionary dynamics. Results used the members-recruit group-formation model, where $h \equiv 1 - q$, with the same parameter values as Fig. 3: $n = 8$, $W = 2$, $Y = 3$, $Z = 0$; (a) $\tau = 5$, $X = -1$; (b) $\tau = 4$, $X = -0.5$. Coloured regions separate regions of qualitatively different evolutionary dynamics: $h < \hat{h}$, Cooperators cannot persist (dark shading); $h < h_1$, Defectors can both invade and persist (red shading); $h > h_0$, Cooperators can invade (blue shading).

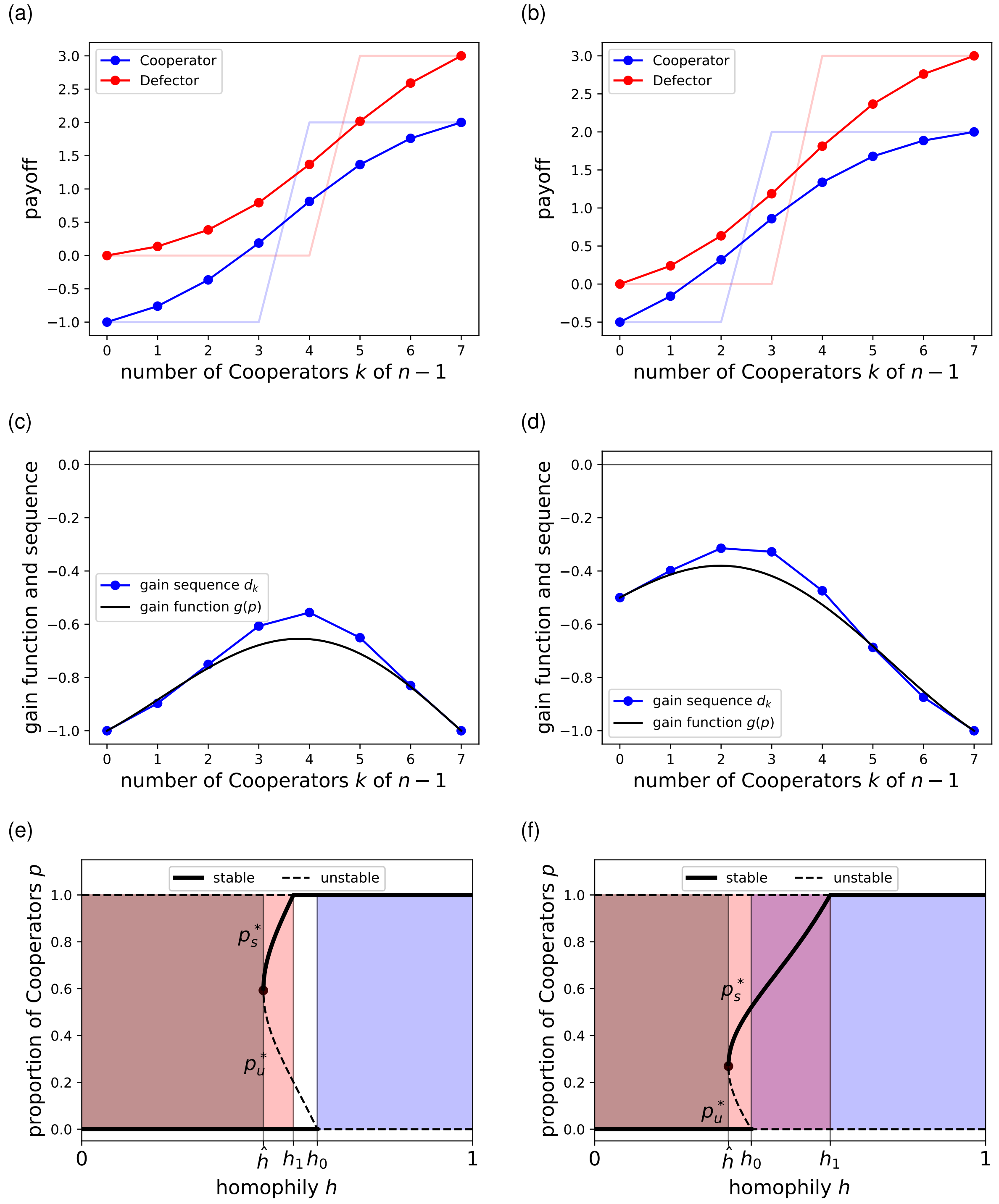

Figure S4: For comparison with Fig. 3 in the main text, two examples of how homophily affects the evolutionary dynamics in the sigmoid game with $s = 5$. (a-b) Cooperator and Defector payoff functions, with the threshold-game functions for comparison with opaque colours; (c-d) Gain sequence and gain function; and (e-f) summary of evolutionary dynamics. Results used the members-recruit group-formation model, where $h \equiv 1 - q$, with the same parameter values as Fig. 3: $n = 8$, $W = 2$, $Y = 3$, $Z = 0$; (a) $\tau = 5$, $X = -1$; (b) $\tau = 4$, $X = -0.5$. Coloured regions separate regions of qualitatively different evolutionary dynamics: $h < \hat{h}$, Cooperators cannot persist (dark shading); $h < h_1$, Defectors can both invade and persist (red shading); $h > h_0$, Cooperators can invade (blue shading).

S6 Critical homophily levels defining different regimes of the evolutionary dynamics

The critical homophily level h_0 separates the region where Cooperators can invade a population of Defectors from where they cannot (e.g., Fig. 3). Therefore, h_0 solves the point where the Cooperator invasion fitness is zero

$$\lim_{p\to 0} \frac{\Delta p}{p} = 0. \tag{S33}$$

The higher-order genetic association indices are a function of the homophily level, $\theta_{l\to m}(h)$, and

$$\rho_l = \rho_l(p,h) = \begin{cases} 1 & \text{if } l = 0, \\ \sum_{m=1}^{l} \theta_{l\to m}(h) p^m & \text{otherwise.} \end{cases} \tag{S34}$$

Therefore,

$$\lim_{p\to 0} \frac{(1-\rho_1)\rho_{l+1}}{p} = \lim_{p\to 0} \frac{(1-p)\sum_{m=1}^{l+1} \theta_{l+1\to m}(h) p^m}{p} = \theta_{l+1\to 1}(h), \quad \text{for } l \geq 0. \tag{S35}$$

and

$$\lim_{p\to 0} \frac{\rho_1(\rho_l - \rho_{l+1})}{p} = \lim_{p\to 0} \frac{p(\rho_l - \rho_{l+1})}{p} = \begin{cases} 1 & \text{if } l = 0, \\ 0 & \text{otherwise.} \end{cases} \tag{S36}$$

Applying the invasion fitness criterion Eq. S33 along with Eqs. S35 and S36 to Eq. S2, h_0 solves

$$\begin{aligned} 0 &= \lim_{p\to 0} \frac{\Delta p}{p}, \\ &= \underbrace{\sum_{k=0}^{n-1}\sum_{l=k}^{n-1} (-1)^{l-k} \binom{l}{k}\binom{n-1}{l} \left[\lim_{p\to 0} \frac{(1-\rho_1)\rho_{l+1}}{p} a_k\right]}_{\text{LHS}} - \underbrace{\sum_{k=0}^{n-1}\sum_{l=k}^{n-1} (-1)^{l-k} \binom{l}{k}\binom{n-1}{l} \left[\lim_{p\to 0} \frac{\rho_1(\rho_l - \rho_{l+1})}{p} b_k\right]}_{\text{RHS}} \end{aligned} \tag{S37}$$

Substituting Eq. S34 into the right-hand term, the sum term only has one term, when $k = 0$ and $l = 0$

$$\begin{aligned} \text{RHS} &= \sum_{k=0}^{n-1}\sum_{l=k}^{n-1} (-1)^{l-k} \binom{l}{k}\binom{n-1}{l} \left[\lim_{p\to 0} \frac{\rho_1(\rho_l - \rho_{l+1})}{p} b_k\right], \\ &= (-1)^0 \binom{0}{0}\binom{n-1}{0} b_0, \\ &= b_0. \end{aligned} \tag{S38}$$

Substitute in the payoffs to Defectors in the threshold game

$$b_k = \begin{cases} Y & \text{if } k \geq \tau, \\ Z & \text{otherwise,} \end{cases} \tag{S39}$$

and recall that we assume $\tau > 1$, then

$$\text{RHS} = Z. \tag{S40}$$

Substitute Eq. S35 into the left-hand term

$$\begin{aligned}\text{LHS} &= \sum_{k=0}^{n-1}\sum_{l=k}^{n-1}(-1)^{l-k}\binom{l}{k}\binom{n-1}{l}\left[\lim_{p\to 0}\frac{(1-\rho_1)\rho_{l+1}}{p}a_k\right],\\ &= \sum_{k=0}^{n-1}\sum_{l=k}^{n-1}(-1)^{l-k}\binom{l}{k}\binom{n-1}{l}\theta_{l+1\to 1}(h_0)\,a_k.\end{aligned}$$

The indices can be rearranged according to the pattern

$$\sum_{k=0}^{n-1}\sum_{l=k}^{n-1}f(k,l)=\sum_{l=0}^{n-1}\sum_{k=0}^{l}f(k,l).$$

Therefore

$$\begin{aligned}\text{LHS} &= \sum_{l=0}^{n-1}\sum_{k=0}^{l}(-1)^{l-k}\binom{l}{k}\binom{n-1}{l}\theta_{l+1\to 1}(h_0)\,a_k,\\ &= \sum_{l=0}^{n-1}(-1)^{l}\binom{n-1}{l}\theta_{l+1\to 1}(h_0)\left[\sum_{k=0}^{l}(-1)^{k}\binom{l}{k}a_k\right].\end{aligned} \tag{S41}$$

Substitute in the payoffs to Cooperators in the threshold game

$$a_k=\begin{cases}W & \text{if } k\geq \tau-1,\\ X & \text{otherwise,}\end{cases}$$

and separate out the cases

$$\begin{aligned}\text{LHS} &= \sum_{l=0}^{\tau-2}(-1)^{l}\binom{n-1}{l}\theta_{l+1\to 1}(h_0)\left[X\sum_{k=0}^{l}(-1)^{k}\binom{l}{k}\right]\\ &\quad+\sum_{l=\tau-1}^{n-1}(-1)^{l}\binom{n-1}{l}\theta_{l+1\to 1}(h_0)\left(\left[X\sum_{k=0}^{\tau-2}(-1)^{k}\binom{l}{k}\right]+\left[W\sum_{k=\tau-1}^{l}(-1)^{k}\binom{l}{k}\right]\right),\\ &= \sum_{l=0}^{\tau-2}(-1)^{l}\binom{n-1}{l}\theta_{l+1\to 1}(h_0)\left[X\sum_{k=0}^{l}(-1)^{k}\binom{l}{k}\right]\\ &\quad+\sum_{l=\tau-1}^{n-1}(-1)^{l}\binom{n-1}{l}\theta_{l+1\to 1}(h_0)\left(\left[X\sum_{k=0}^{l}(-1)^{k}\binom{l}{k}\right]+\left[(W-X)\sum_{k=\tau-1}^{l}(-1)^{k}\binom{l}{k}\right]\right).\end{aligned} \tag{S42}$$

Into Eq. S42, substitute two identities, a definition, and an assumption about the model. The two identities are

$$\sum_{k=0}^{l}(-1)^{k}\binom{l}{k}=\begin{cases}1 & \text{if } l=0,\\ 0 & \text{if } l>0,\end{cases}$$

and

$$\sum_{k=D}^{l}(-1)^{k}\binom{l}{k}=(-1)^{D}\binom{l-1}{D-1}.$$

The definition is

$$\theta_{1\to 1}=1.$$

The model assumption is that the number of cooperators needed to achieve the public good is greater than 1, $\tau>1$, which implies

$$\tau-1>0.$$

Substituting these four items into Eq. S42, the left-hand term simplifies to

$$\text{LHS} = X + (W - X) \sum_{l=\tau-1}^{n-1} (-1)^{l-\tau+1} \binom{n-1}{l} \binom{l-1}{\tau-2} \theta_{l+1\to 1}(h_0). \tag{S43}$$

Bringing Eqs. S40 and S43 together, h_0 solves

$$\begin{aligned} 0 &= \text{LHS} - \text{RHS}, \\ 0 &= X - Z + (W - X) \sum_{l=\tau-1}^{n-1} (-1)^{l-\tau+1} \binom{n-1}{l} \binom{l-1}{\tau-2} \theta_{l+1\to 1}(h_0). \end{aligned} \tag{S44}$$

By similar reasoning, the critical homophily level h_1, which separates the region where Defectors can invade a population of Cooperators from where they cannot, solves

$$0 = Y - W + (Z - Y) \sum_{l=n-\tau}^{n-1} (-1)^{l-n+\tau} \binom{n-1}{l} \binom{l-1}{n-\tau-1} \theta_{l+1\to 1}(h_1). \tag{S45}$$

Eqs. S44 and S45 provide analytical descriptions; however, given the complexity of the relationships between the genetic association indices and homophily, the quantities h_0 and h_1 were solved numerically. To solve for $\hat{h}$, we also used a numerical approach; see scripts `calc_isocline.py`.

S7 The effects of group size and threshold

Increasing the group size n has a negative effect on cooperation (Fig. S5a,c,e) by increasing the degree of homophily needed for Cooperators to invade and persist. Beyond a certain group size (e.g., $n = 8, 10$ in Fig. S5a), Cooperators cannot persist without homophilic group formation.

Threshold τ has a mixed effect on cooperation (Fig. S5b,d,f). High thresholds prevent Cooperators from invading unless homophily is strong. However, high thresholds also prevent invasion by Defectors unless homophily is weak and increase the proportion of Cooperators in the polymorphic population. Choosing an intermediate threshold level can cause the loss of the polymorphic coexistence between Cooperators and Defectors. However, the polymorphic equilibrium can be regained by increasing homophily. Increasing homophily also moves the polymorphic equilibrium away from all-Defectors equilibrium, reducing the risk of extinction of Cooperators under stochastic dynamics in finite populations [cf., 132].

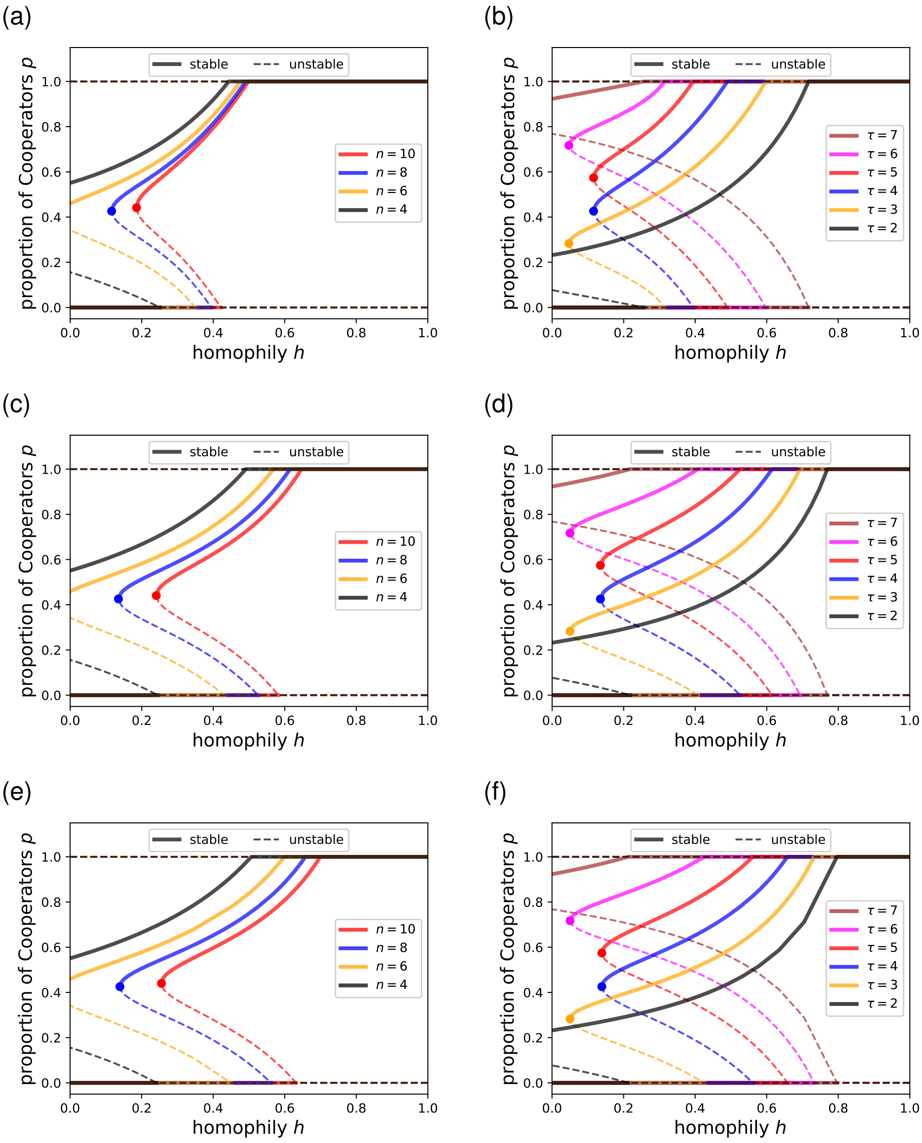

Figure S5: Examples of how the evolutionary dynamics are affected by (a,c,e) group size n (threshold proportion constant, $\tau/n = 1/2$), and (b,d,e) threshold τ (group size constant, $n = 8$), in the (a,b) leader-driven, (c,d) member-recruits, and (e,f) member-attracts group-formation models. Parameters: $W = 2$, $X = -1$, $Y = 3$, and $Z = 0$.

S8 Family abundance distribution when the group size is large

Fig. S6 compares how evolutionary dynamics under the three group-formation models change with group size n (where threshold is kept at fixed proportion $\tau = n/2$) for the default parameter values used in the main text. The leader-driven group-formation model is the most favourable to cooperation, particularly at larger group sizes. In the member-driven models, each new nonkin member has a chance to itself become a recruitor or to become the one who attracts a new member, the new member being one of its own family members, potentially another Defector; whereas recruitments of nonkin do not propagate in the leader-driven model.

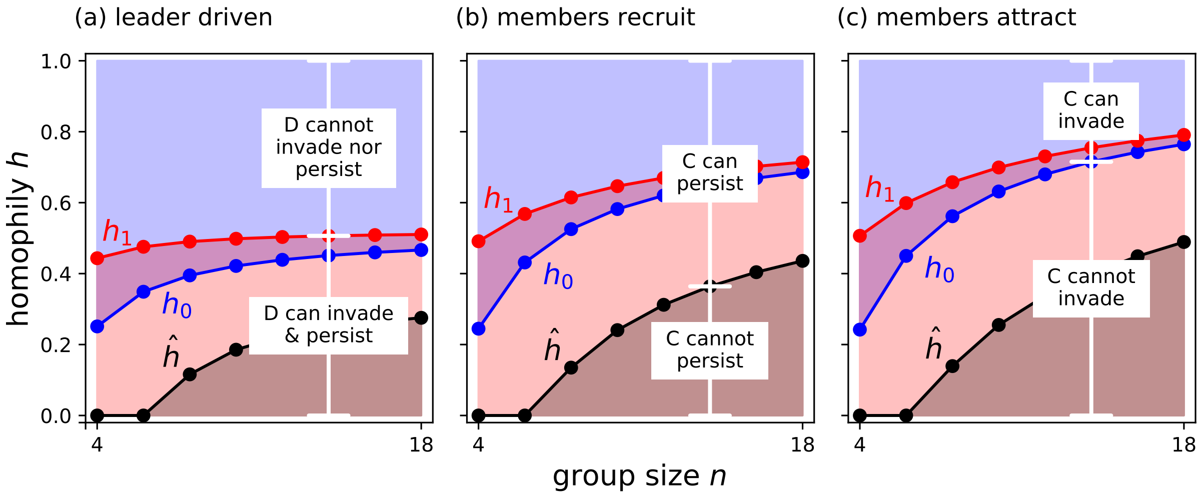

Figure S6: A comparison of the dynamics between different group-formation models: (a) leader driven, (b) members recruit, and (c) members attract. Threshold $\tau = n/2$ held constant, $W = 2$, $X = -1$, $Y = 3$, $Z = 0$. Coloured regions correspond to those in Fig. 3: $h < \hat{h}$, Cooperators cannot persist (dark shading); $h < h_1$, Defectors can both invade and persist (red shading); $h > h_0$, Cooperators can invade (blue shading). The two text boxes on each panel are not specific to a panel, but rather the six text boxes apply to all three panels.

The comparison in Fig. S6 is limited to the group-size range shown because of the combinatorial nature of the calculations that must be made to calculate the probabilities of different family partition structures (details in S2 and S3). However, we can still compare the effects of the three group-formation models by exploring how the expected proportion of individuals in the largest family groups changes with group size (e.g., Fig. S7).

In the leader-driven group-formation model, as n increases, the proportion of individuals in the largest family group (i.e., the leader's) approaches $1 - q$, and all others (singleton families) approach 0 (Fig. S7a). This follows directly from the definition of the leader-driven model, that the probability of recruiting an individual who is not a member of the leader's family is q.

In the members-recruit group-formation model, the probability of recruiting a nonkin member is a constant q. Therefore, as n increases, the proportion of individuals in the largest family groups asymptotes to zero (Fig. S7b).

In the members-attract group-formation model, as n increases, the family abundance distribution is known to converge to a stable relative abundance distribution [e.g., Chap. 5, Fig. 5.3, 133] (Fig. S7c). The intuitive reason is because, as the number of individuals j in the group increases, the probability of recruiting a nonkin member at the next recruitment approaches zero

$$\lim_{j\to\infty} P(j \text{ from a new family}) = \lim_{j\to\infty} \frac{\alpha}{\alpha + j} = 0. \tag{S46}$$

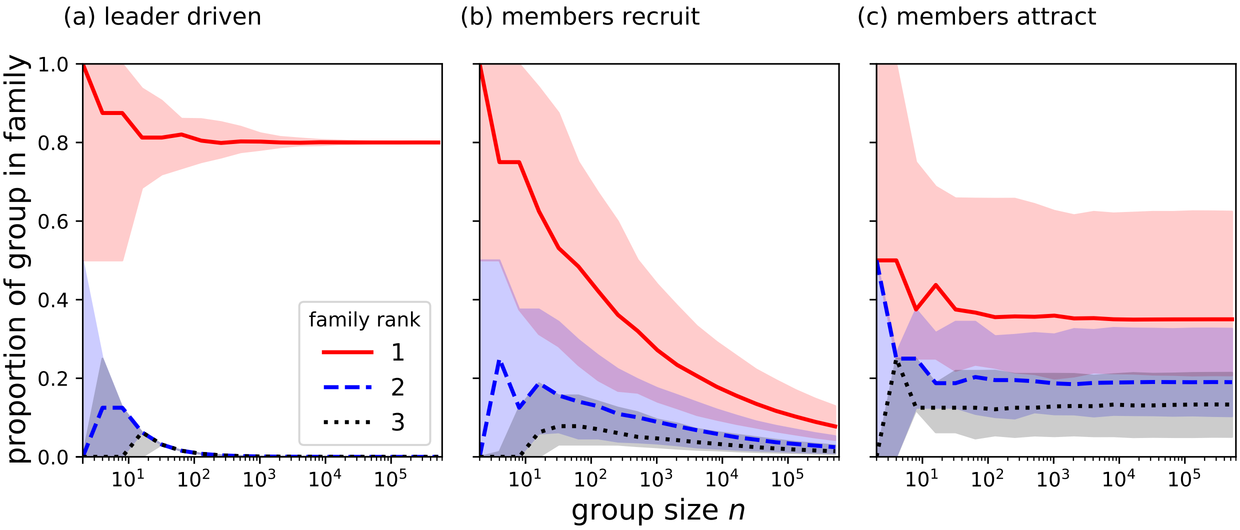

Figure S7: An example of how the proportion of individuals from the three largest families in the group (rank 1, 2, and 3) changes with group size n for different group-formation models: (a) leader driven, $q = 0.2$; (b) members recruit, $q = 0.2$; and (c) members attract, $\alpha = 3$. The example was generated by running one sequential sampling scheme for each model (`/scripts/family_abundance_distn/calc_relative_abundances.py`).

S9 Overview of the code repository

Functions and scripts used to generate the results are available from the Github repository `https://github.com/nadiahpk/homophilic-threshold-PGG`, which is also archived at DOI: 10.5281/zenodo.7344398.

A quickstart tutorial to calculate Δp can be found in:
`/tutorials/calculate_deltap.pdf`.

Matrix M used to calculate $\boldsymbol{\theta}$ from $\mathbf{F}$ is calculated using the the script:
`/scripts/matrix_M/save_matrix_Ms.py`.

Precalculated M matrices up to size $n = 24$ are stored in `/results/matrix_M/` and can be read using `read_matrix_M()` in `/functions/my_functions.py`.

A quickstart tutorial for how to use `read_matrix_M()` is provided in:
`/tutorials/matrix_M.pdf`.

The combinatorial term needed to calculate family partition structure probabilities for the members-recruit group-formation model (i.e., $\sum_{\vec{\mathbf{n}}} \sum_{\mathbf{m}} C(\mathbf{m}, \vec{\mathbf{n}}) \prod_k m_k$) can be calculated using the script:
`scripts/members_recruit/sum_product_mistakes/save_sum_prod_mistakes.py`.

Precalculated terms up to size $n = 18$ are stored in:
`/results/members_recruit/sum_product_mistakes/`.

Supplementary references

[120] Ajar, É. 2003 Analysis of disruptive selection in subdivided populations. *BMC Evolutionary Biology*, **3**(1), 1–12.

[121] Roze, D. & Rousset, F. 2008 Multilocus models in the infinite island model of population structure. *Theoretical Population Biology*, **73**(4), 529–542.

[122] Gardner, A. & West, S. A. 2010 Greenbeards. *Evolution: International Journal of Organic Evolution*, **64**(1), 25–38.

[123] Taylor, P. 2013 Inclusive and personal fitness in synergistic evolutionary games on graphs. *Journal of Theoretical Biology*, **325**, 76–82.

[124] Wakano, J. Y. & Lehmann, L. 2014 Evolutionary branching in deme-structured populations. *Journal of Theoretical Biology*, **351**, 83–95.

[125] Mullon, C., Keller, L. & Lehmann, L. 2016 Evolutionary stability of jointly evolving traits in subdivided populations. *The American Naturalist*, **188**(2), 175–195.

[126] Mullon, C., Keller, L. & Lehmann, L. 2018 Social polymorphism is favoured by the co-evolution of dispersal with social behaviour. *Nature Ecology & Evolution*, **2**(1), 132–140.

[127] Mullon, C. & Lehmann, L. 2019 An evolutionary quantitative genetics model for phenotypic (co) variances under limited dispersal, with an application to socially synergistic traits. *Evolution*, **73**(9), 1695–1728.

[128] Parvinen, K., Ohtsuki, H. & Wakano, J. Y. 2020 Evolution of dispersal in a spatially heterogeneous population with finite patch sizes. *Proceedings of the National Academy of Sciences*, **117**(13), 7290–7295.

[129] Donnelly, P. 1986 Partition structures, Polya urns, the Ewens sampling formula, and the ages of alleles. *Theoretical Population Biology*, **30**(2), 271–288.

[130] Kelleher, J. & O'Sullivan, B. 2009 Generating all partitions: a comparison of two encodings. *arXiv preprint arXiv:0909.2331*.

[131] Hofbauer, J. & Sigmund, K. 1998 *Evolutionary games and population dynamics*. Cambridge: Cambridge University Press.

[132] Tutić, A. 2021 Stochastic evolutionary dynamics in the volunteer's dilemma. *The Journal of Mathematical Sociology*, pp. 1–20.

[133] Hubbell, S. P. 2001 *The unified neutral theory of biodiversity and biogeography*, vol. 32. Princeton, USA: Princeton University Press.
